# Adaptive Online Sequential ELM for Concept Drift Tackling

**DOI:** 10.1155/2016/8091267

**Published:** 2016-08-09

**Authors:** Arif Budiman, Mohamad Ivan Fanany, Chan Basaruddin

**Affiliations:** Faculty of Computer Science, University of Indonesia, Depok, West Java 16424, Indonesia

## Abstract

A machine learning method needs to adapt to over time changes in the environment. Such changes are known as concept drift. In this paper, we propose concept drift tackling method as an enhancement of Online Sequential Extreme Learning Machine (OS-ELM) and Constructive Enhancement OS-ELM (CEOS-ELM) by adding adaptive capability for classification and regression problem. The scheme is named as adaptive OS-ELM (AOS-ELM). It is a single classifier scheme that works well to handle real drift, virtual drift, and hybrid drift. The AOS-ELM also works well for sudden drift and recurrent context change type. The scheme is a simple unified method implemented in simple lines of code. We evaluated AOS-ELM on regression and classification problem by using concept drift public data set (SEA and STAGGER) and other public data sets such as MNIST, USPS, and IDS. Experiments show that our method gives higher kappa value compared to the multiclassifier ELM ensemble. Even though AOS-ELM in practice does not need hidden nodes increase, we address some issues related to the increasing of the hidden nodes such as error condition and rank values. We propose taking the rank of the pseudoinverse matrix as an indicator parameter to detect “underfitting” condition.

## 1. Introduction

Data stream mining is a data mining technique, in which the trained model is updated whenever new data arrive. However, the trained model must work in dynamic environments, where a vast amount of data not only is continuously generated but also keeps changing. This challenging issue is known as concept drift [1], in which the statistical properties of the input attributes and target classes shifted over time. Such shifts can make the trained model less accurate.

More methods for concept drift handling can be found in the literature [1], where the aim is to boost the generalization accuracy. These methods pursue an accurate, simple, fast, and flexible way to retain classification performance when the drift occurs. Ensemble classifier is a well-known way to retain the classification performance. The combined decision of many single classifiers (mainly using ensemble members diversification) is more accurate than single classifier [2]. However, it has higher complexity when handling multiple (consecutive) concept drifts.

One of the popular machine learning methods is Extreme Learning Machine (ELM) introduced by Huang et al. [3–7]. The ELM is a Single-Layer Feedforward Neural Network (SLFN) with fast learning speed and good generalization capability.

In this paper, we focused on the learning adaptation method as an enhancement to Online Sequential Extreme Learning Machine (OS-ELM) [8] and Constructive Enhancement OS-ELM (CEOS-ELM) [9]. We named it as adaptive OS-ELM (AOS-ELM). The AOS-ELM has capability to handle multiple concept drift problems, either changes in the number of attributes (virtual drift/VD) or the number of target classes (real drift/RD) or both at the same time (hybrid drift/HD), also for recurrent context (all concepts occur alternately) or sudden drift (new concept substitutes previous concepts) [10]. Our scope of attribute changes discussed in this paper is on the feature space concatenation widely used in data fusion, kernel fusion, and ensemble learning [11] and not on the feature selection (irrelevant features removal) methods [12]. We compared the performance with nonadaptive sequential ELM: OS-ELM and CEOS-ELM. We also compared the performance with ELM classifier ensembles as the common adaptive approach for concept drift solution. In the present study, although we focus on the adaptation aspect, we address some possible change detection mechanisms that are suitable for our method.

A preliminary version of RD and its early results appeared in conference proceedings [14]. In this paper, we introduced the new scenarios in VD, HD, and consecutive drifts, either recurrent or sudden drift scenarios as well as theoretical background explanation. Our main contributions in this research area can be summarized as follows:

1. We proposed simple adaptive method as enhancement to OS-ELM and CEOS-ELM for addressing concept drifts issue. Unlike ensemble systems [6, 13] that need to manage the complex combination of a vast number of classifiers, we pursue a single classifier for simple implementation while retaining comparable performance for handling multiple (consecutive) drifts.
2. We introduced a simple unified platform to handle a hybrid drift (HD) when changes in the number of attributes and the number of target classes occurred at the same time.
3. We elaborated how the AOS-ELM for transfer learning uses hybrid drift strategy. Transfer learning focuses on extracting the knowledge from one or more source task domains and applies the knowledge to a different target task domain [15]. Concept drift focuses on the time-varying domain with a small number of current data available. In contrast, transfer learning is not associated with time and requires the entire training and testing data set [16]. The example of transfer learning by using HD strategy is the transition from different data set sources but still related and with the same purpose. In this paper, we discussed the transfer learning on numeric handwritten MNIST [17] to alphanumeric handwritten USPS [18] recognition.
4. Naturally, the AOS-ELM handling strategy was based on recurrent context. We devised an AOS-ELM strategy to handle sudden drift scenario by introducing output marginalization method. This method is also applicable for concept drift in a regression problem.
5. We studied the effect of increasing the number of hidden nodes, which is treated as one of learning parameters, to improve the accuracy (other learning parameters are input weight, bias, activation function, and regularization factor). We proposed the evaluation parameter to predict the accuracy before the training was completed. We applied this assessment parameter actually to prevent “underfitting” or nonconvergence condition (the model does not fit the data well enough that makes accuracy performance dropped) when any learning parameter changes such as hidden nodes increased.

This paper is organized as follows.  Section 2 explains some issues and challenges in concept drift, the background of ELM, and ELM in sequential learning.  Section 3 presents the background theory and algorithm derivation of the proposed method. In Section 4, we focus on the empirical experiments to prove the methods and research questions in regression and classification problem. We use artificial and real data set. The artificial data sets are streaming ensemble algorithm (SEA) [19] and STAGGER [20], which are commonly used as benchmark in sequential learning. The real data sets are handwritten recognition data: MNIST for numeric [17] and USPS for alphanumeric classes [18]. We studied the effect of hidden nodes increase as one of the important learning parameters in Section 4.5.  Section 7 discusses research challenges and future directions. The conclusion presents some highlights in Section 8.

## 2. Related Works

### 2.1. Notations

We specify the notations used throughout this article for easier understanding as follows:

(i) Matrix is written in uppercase bold (e.g., **X**).
(ii) Vector is written in lowercase bold (e.g., **x**).
(iii) The transpose of a matrix **X** is written as **X** ^T^. The pseudoinverse of a matrix **H** is written as **H** ^†^.
(iv) *f*, *g* will be used as nonlinear differentiable function (activation function), for example, sigmoid or tanh function.
(v) The amount of training data is *N*. Each input data **x** contains some *d* attributes. The target has *m* number of classes. An input matrix **X** can be denoted as **X**_*d*×*N*_ and the target matrix **T** as **T**_*N*×*m*_.
(vi) The hidden layer matrix is **H**. The input weight matrix is **A**. The output weight matrix is **β**. The matrix Δ**H** is the additional block portion of the matrix **H**. The matrix **K** is the autocorrelation matrix of **H** ^T^ **H**. The inverse of matrix **K** is **P**.
(vii) **H** can be denoted as **H**_*N*×*L*_. **A** can be denoted as **A**_*d*×*L*_ and **β** can be denoted as **β**_*L*×*m*_. *δL* denotes the additional nodes number of *L*.
(viii) When the number of training data *N* → *∞*, we employed the online sequential learning method by updating model every time each new training pairs (**x**, **t**) are seen. **X**_(0)_ is the subset of input data at time *k* = 0 as the initialization stage. **X**_(1)_, **X**_(2)_,…, **X**_(*k*)_ are the subset of input data at the next sequential time. Each subset may have different number of quantities. The corresponding label data is presented as **T** = [**T**_(0)_, **T**_(1)_, **T**_(2)_,…, **T**_(k)_]. We used the subscript font with parenthesis to show the sequence number.
(ix) We denote the training data from different *S* concepts (sources or contexts), using the symbol **X**_*s*_ for training data and **T**_*s*_ for target data. We used the subscript font without parenthesis to show the source number.
(x) We denote the drift event using the symbol  _VD_^⋙^, where the subscript font shows the drift type. For example, Concept 1 has virtual drift event to be replaced by Concept 2 (sudden drift): **C**_1__VD_^⋙^ **C**_2_. Concept 1 has real drift event to be replaced by Concept 1 and Concept 2 recurrently (recurrent context) in the shuffled composition: **C**_1__RD_^⋙^ shuffled(**C**_1_, **C**_2_).

### 2.2. Concept Drift Strategies

In this section, we briefly explained the various concept drift solution strategies.

Gama et al. [1] explained that many concept drift methods have been developed, but the terminologies are not well established. According to Gama et al., the basic concept drift based on Bayesian decision theory in the classification problem for class output *c* and incoming data **X** is

(1) $$\begin{array}{c} P\left(\;c\;|\;X\;\right)=P\left(\;c\;\right)\frac{P\left(X\;|\;c\right)}{P\left(X\right)}. \end{array}$$

Concept drift occurred when *P*(*c*∣**X**) has changed; for example, ∃**X** : *P*_(0)_(**X**, *c*) ≠ *P*_(1)_(**X**, *c*), where *P*_(0)_ and *P*_(1)_ are, respectively, the joint distribution at times *t*_(0)_ and *t*_(1)_. Gama et al. categorized the concept drift types as follows:

(1) Real drift (RD) refers to changes in *P*(*c*∣**X**). The change in *P*(*c*∣**X**) may be caused by a change in the class boundary (the number of classes) or the class conditional probabilities (likelihood) *P*(**X**∣*c*). The number of classes expanded and different class of data may come alternately, known as recurrent context. A drift, where new conditional probabilities replace the previous conditional probabilities while the number of classes remained the same, is known as sudden drift. Other terms are concept shift or conditional change [21].
(2) Virtual drift (VD) refers to the changes in the distribution of the incoming data (e.g., *P*(**X**) changes). These changes may be due to incomplete or partial feature representation of the current data distribution. The trained model is built with additional data from the same environment without overlapping the true class boundaries. Other terms are feature change [21], temporary drift, or sampling shift.

Kuncheva [10, 22] explained the various configuration patterns of data sources over time as random noise, random trends (gradual changes), random substitutions (abrupt or sudden changes), and systematic trends (recurring context). The random noise will simply be filtered out. A gradual drift occurs when many concepts may reoccur alternately in the gradual stage for a certain period. A consecutive drift takes place when many previously active concepts might keep on changing alternately (recurring context) after some time. The sudden drift (abrupt changes or concept substitutions) is the type that at one time one concept is suddenly replaced by another concept.

**Figure 1:**
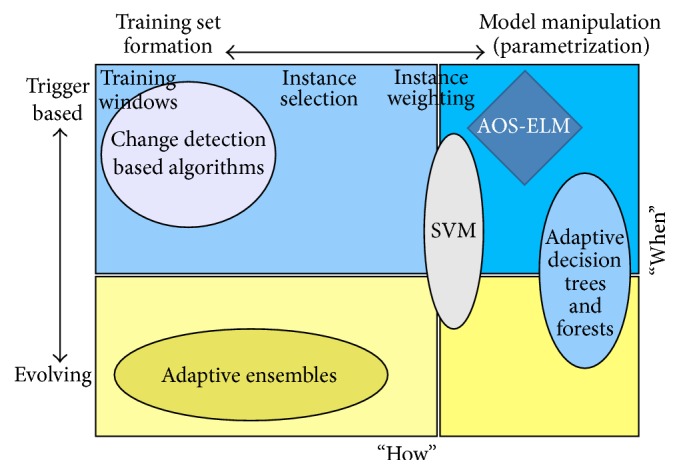
The taxonomy quadrant of adaptive supervised learning techniques. Popular concept drift handling methods are indicated by ellipses [13]. Our proposed method AOS-ELM is indicated by a dark blue diamond.

Žliobaitė [13] proposed a taxonomy of concept drift tackling methods as shown in Figure 1. It describes the methods based on when the model is switched on (the “when” axis) and how the learners adapt to training set formation or design and parametrization of the base learner (the “how” axis). The “when” axis spans drift handling from trigger based to evolving based methods. The “how” axis spans drift handling from training set formation to model manipulation (or parametrization) methods.

Žliobaitė [13] explained that most attention on the concept drift tackling methods is drawn to multiclassifier model selection and fusion rules, but little attention is drawn on the model construction of base classifier.

**Figure 2:**
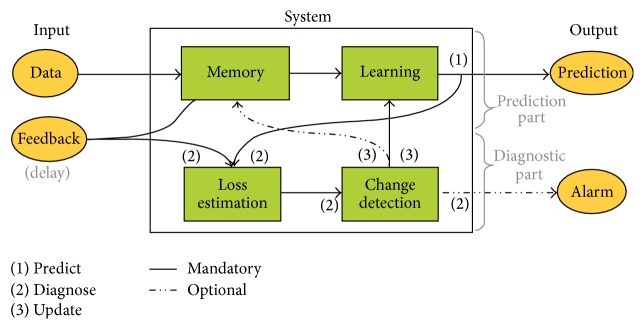
A generic scheme for an online adaptive learning algorithm from Gama et al. [1].

Gama et al. [1] proposed a complete online adaptive learning scheme that organized four modules: memory, change detection, learning, and loss estimation (see Figure 2). These modular components can be integrated, permuted, and combined with each other. The key modules are the learning and the change detection modules. Most methods focused on some subset or often mixtures of many types within certain concept drifts.

The learning module refers to the methods for the adaptation strategies of the predictive model. The learning module is categorized based on (i) how the model is updated when new data points are available (learning mode): retraining or incremental (online) modes; (ii) the behavior of predictive models on time-evolving data (model adaptation): a blind (evolving or implicit) based module or an informed (trigger or explicit) based module; (iii) the techniques for maintaining active predictive models (model management): a single model or ensemble model. The change detection module refers to drift detection. The change detection identifies change points or small time intervals when changes occur.

Each drift employed different solution strategies. The solution for RD is entirely different from VD. If the systematic changes are likely to reappear, we may want to keep past successful classifiers and simply reuse them. If the changes are gradual, we may use a moving window strategy on the training data. If the changes are abrupt, we can pause the existing static classifiers and then retrain the classifier using the new training data. Thus, it is hard to combine simultaneously many strategies at one time to solve many types of concept drift in just a simple platform.

### 2.3. ELM in Sequential Learning

In this section, we briefly explained the previous related works of ELM in sequential learning and adaptive environments.

ELM is getting popularity thanks to its learning speed, generalization capability, and simplicity. Huang [5] explained the term “Extreme” meant to move beyond conventional artificial neural network learning that required iterative tuning. The ELM moves toward brain-like learning in which hidden neurons need not be tuned.

The output function of an SLFN with single hidden layer matrix **H** can be presented as the function of

(2) $$\begin{array}{c} f_{L}\left(\;x\;\right)=\overset{L}{\underset{i=1}{\sum }}\beta_{i}H\left(\;a_{i},b_{i},x\;\right), \end{array}$$

where **H** = *g*(**A** **X** + **b**). All of these parameters defining the values of **H** elements are named as hidden node parameters [6].

The solution of ELM training with the smallest error can be obtained when the output weight **β** is approximated by

(3) $$\begin{array}{c} \hat{\beta }=H^{†}T, \end{array}$$

where **H** ^†^ is the pseudoinverse of **H**.

**H** ^†^ can be approximated by left pseudoinverse of **H** as

(4) $$\begin{array}{c} \hat{\beta }={\left(\;H^{T}H\;\right)}^{-1}H^{T}T. \end{array}$$

We can use ridge regression or regularized least squares to be $\hat{\beta }=(H^{T}H+I/c{)}^{-1}H^{T}T$.

Based on [4], Liang et al. [8] proposed online learning for ELM named OS-ELM. If we have ${\hat{\beta }}_{\left(0\right)}$ from **H**_(0)_ filled by the *N*_0_ number of training data and *N*_1_ incremental batch of data filled **H**_(1)_, the output weights ${\hat{\beta }}_{\left(1\right)}$ are approximated by

(5) $$\begin{array}{c} {\hat{\beta }}_{\left(1\right)}={\left(\;{\left[\;\begin{array}{c} H_{\left(0\right)}\\ H_{\left(1\right)} \end{array}\;\right]}^{T}\left[\begin{array}{c} H_{\left(0\right)}\\ H_{\left(1\right)} \end{array}\right]\;\right)}^{-1}{\left[\;\begin{array}{c} H_{\left(0\right)}\\ H_{\left(1\right)} \end{array}\;\right]}^{T}\left[\begin{array}{c} T_{\left(0\right)}\\ T_{\left(1\right)} \end{array}\right]. \end{array}$$

Both **H**_0_ and **H**_1_ have a different number of training data but have the same *L* number of hidden nodes.

If **K** = **H** ^T^ **H**, then we can rewrite

(6) $$\begin{array}{c} {\hat{\beta }}_{\left(1\right)}=K_{\left(1\right)}^{-1}{\left[\;\begin{array}{c} H_{\left(0\right)}\\ H_{\left(1\right)} \end{array}\;\right]}^{T}\left[\begin{array}{c} T_{\left(0\right)}\\ T_{\left(1\right)} \end{array}\right]. \end{array}$$

The OS-ELM assumes no changes in the number of hidden nodes. However, increasing the number of hidden nodes is required to improve the performance. A CEOS-ELM [9] has addressed this problem by adding hidden nodes in the sequential learning stage. So $H=\left[\begin{array}{cc} H_{\left(0\right)} & \Delta H_{\left(0\right)}\\ H_{\left(1\right)} & \Delta H_{\left(1\right)} \end{array}\right]$. The submatrix Δ**H**_(0)_ is set to a zero block matrix to simplify the computation in accordance with the fact that the previous data is not related to the new hidden nodes. The additional hidden nodes block matrix Δ**H**_(1)_ for *N*_1_ data has relation to the additional hidden nodes *δL*_(1)_.

Then, we can rewrite **K**_(1)_ with Δ**H**_(1)_ as

(7) $$\begin{array}{c} {\hat{K}}_{\left(1\right)}={\left[\;\begin{array}{cc} H_{\left(0\right)} & 0\\ H_{\left(1\right)} & \Delta H_{\left(1\right)} \end{array}\;\right]}^{T}\left[\begin{array}{cc} H_{\left(0\right)} & 0\\ H_{\left(1\right)} & \Delta H_{\left(1\right)} \end{array}\right]. \end{array}$$

If $\hat{P}={\hat{K}}^{-1}$ can be solved using block matrix inversion and Schur complement, then

(8) $$\begin{array}{c} {\hat{\beta }}_{\left(1\right)}={\hat{P}}_{\left(1\right)}{\left[\;\begin{array}{cc} H_{\left(0\right)} & 0\\ H_{\left(1\right)} & \Delta H_{\left(1\right)} \end{array}\;\right]}^{T}\left[\begin{array}{c} T_{\left(0\right)}\\ T_{\left(1\right)} \end{array}\right]. \end{array}$$

It is important to note that both OS-ELM and CEOS-ELM did not address the concept drift issue; for example, when the number of attributes *d* in **X**_*d*×*N*_ or the number of classes *m* in **T**_*N*×*m*_ in data set has been added. In this paper, we categorized OS-ELM and CEOS-ELM as nonadaptive sequential ELM.

To the best of our knowledge, no previous single base ELM approach specifically addresses many concept drifts learning [6]. However, some papers [23, 24] already discussed how the ELM is implemented in adaptive environment.

van Schaik and Tapson [23] proposed Online Pseudoinverse Update Method (OPIUM). OPIUM is based on Greville's method as the incremental solutions to compute the pseudoinverse of matrix. The pseudoinverse computation can be solved incrementally as linear regression problems and can be adaptive which allows for nonstationary data. The derivation of OPIUM is equivalent to the OS-ELM if the condition *c*_(*k*)_≝(**I** − **H**_(*k*−1)_ **H**_(*k*−1)_^−1^)**H**_(*k*)_ = 0 is met at each iteration. This condition implies that **H**_(*k*)_ is a linear combination of the previous hidden layer **H**_(*k*−1)_ and the simpler derivation of (3) with right pseudoinverse becoming

(9) $$\begin{array}{c} \hat{\beta }=TH^{†}=TH^{T}{\left(\;H^{T}H\;\right)}^{-1}. \end{array}$$

van Schaik and Tapson defined **ψ** as the cross correlation matrix between **T** and **H** and ***θ*** as the inverse of the autocorrelation **H**, so **β** = **ψ** ***θ***. According to Greville's method, the solution for **ψ**_(*k*)_ = **ψ**_(*k*−1)_ + **T**_(*k*)_ **H**_(*k*)_^T^. And the solution for ***θ***_(*k*)_ = (**H**_(*k*−1)_ **H**_(*k*−1)_^T^ + **H**_(*k*)_ **H**_(*k*)_^T^)^−1^, or in short writing ***θ***_(*k*)_ = *f*(***θ***_(*k*−1)_, **H**_(*k*)_).

van Schaik and Tapson proposed a simplified version named OPIUM light by computing only the on-diagonal element of ***θ***_(*k*)_. van Schaik and Tapson applied the OPIUM light for nonstationary data by using different weight *α* in determining **β**_(*k*)_ for the most recent pair (**T**_(*k*)_, **H**_(*k*)_) appropriate for nonstationary mapping, which are **ψ**_(*k*)_ = (2 − *α*)**ψ**_(*k*−1)_ + *α* **T**_(*k*)_ **H**_(*k*)_^T^ and ***θ***_(*k*)_ = ((2 − *α*)**H**_(*k*−1)_ **H**_(*k*−1)_^T^ + *α* **H**_(*k*)_ **H**_(*k*)_^T^)^−1^.

In our opinion, OPIUM only tackled the real drift case with discriminant function boundary shift in the streaming data (e.g., the frequency shift of sine wave). They implemented the weighting *α* as a nonstationary mapping parameter between input and output vectors.

Cao et al. [24] proposed two-phase classification algorithm: first, weighted ensemble classifier based on ELM (WEC-ELM) algorithm, which can dynamically adjust classifier and the weight of training uncertain data to solve the problem of concept drift, and second, an uncertainty classifier based on ELM (UC-ELM) algorithm designed for the classification of unknown data streams, which considers attribute (tuple) value and its uncertainty, thus improving the efficiency and accuracy. When concept drift occurs, WEC-ELM will dynamically adjust the classifiers and the weight of training data, thus a new classifier will be added to the ensemble until it reached a preset maximum and then removed the worst-performing classifier. UC-ELM is designed for the classification of uncertain data streams, which has attributes (tuples) and its uncertainty values. The UC-ELM evaluated uncertainty value for every newly arrived attribute and decided based on the probability of the new attributes belonging to each class, thus improving the efficiency and accuracy. In our opinion, WEC-ELM is categorized as evolving based method by selecting the best-performing classifier, and UC-ELM addressed virtual drift problem by using uncertainty attributes selection.

Most ELM work in adaptive environments addressed for particular drift case which may be impractical for other cases. We pursue a simple unified platform that has the capability to handle many (consecutive) drift cases.

## 3. Proposed Method

### 3.1. Theoretical Background of AOS-ELM

In sequential learning, some partial training data arrives in time sequential fashion: {(**x**_(0)_, **t**_(0)_), (**x**_(1)_, **t**_(1)_),…, (**x**_(k)_, **t**_(*k*)_)}. Learning is the process of constructing function $\hat{\beta }$ to map between observation and its nature called (class) [25]. When the number of training data *N* → *∞*, we need to address the expected value of $\beta_{\left(\infty \right)}=\hat{\beta }$.

Learning from the data **D**_*n*_ is the process to select a function **β**_*n*_ from a class of *𝔅* by minimizing the empirical squared error *e*_*n*_(**β**) = (1/*n*)∑_*i*=1_^*n*^(**H**_*i*_ **β** − **T**_*i*_)^2^ with the error probability *L*(**β**_*n*_) = *P*{**I**_{**Hβ**_*n*_}_ ≠ **T**∣**D**_*n*_} of the resulting classifier. According to [25], the empirical squared error minimization is consistent under general conditions.

**Theorem 1 .**

Assume that *𝔅* is a totally bounded class of functions. If **β**_*n*_ ∈ *𝔅*, then the classification rule obtained by minimizing the empirical squared error over *𝔅* is strongly consistent; that is,

(10) $$\begin{array}{c} P\left\{\;\underset{n\to \infty }{\lim}L\left(\;\beta_{n}\;\right)=L^{*}\;\right\}⟶1. \end{array}$$

Based on Law of Large Numbers (LLN) theorem [26] and Theorem 1, in sequential learning with the number of training data *N* → *∞*, we can make sure that the consistency of expected value of learning model is $\hat{\beta }=H^{†}T$.

The concept drift refers to an online supervised learning model when the relation between the input data and the target variable changes over time [1]. If the learning model from Concept 1 $\hat{\beta_{1}}\in {𝔅}_{1}$ is bounded by hypothesis space *ℝ* ^*m*_1_^ and feature space *ℝ* ^*d*_1_^ and the learning model from Concept 2 $\hat{\beta_{2}}\in {𝔅}_{2}$ is bounded by hypothesis space *ℝ* ^*m*_2_^ and feature space *ℝ* ^*d*_2_^, we defined the real drift as when the hypothesis space *ℝ* ^*m*_1_^ has changed to *ℝ* ^*m*_2_^. We scoped the definition for *m*_2_ > *m*_1_ dimension changes. The virtual drift is when the feature space *ℝ* ^*d*_1_^ has changed to *ℝ* ^*d*_2_^. We scoped the definition for *d*_2_ > *d*_1_ dimension changes.

To achieve the consistency of minimized square error in the new hypothesis space or new feature space, the learning model needs a transition map from the former space to the new space. The learning model $\hat{\beta_{1}}$ needs a transition space before it converges to the new learning model $\hat{\beta_{2}}\in {𝔅}_{2}\subset {\mathbb{R}}^{m_{2}}$. Our transition space idea was inspired by geometric approach for solving many problems in the fields of pattern recognition and machine learning [27, 28].

For transition space, we propose two approaches: (i) assign the random coordinates in the new concept space and (ii) assign the equivalent projection coordinates in the new design space. The first approach is suitable for VD scenario, in which we assigned the new random coordinates as the new input weight parameters. The second approach is suitable for RD situation, by setting the equivalent projection coordinates in the new space (e.g., (*X*_1_) in 1D coordinate has corresponding 2D projection coordinates as (*X*_1_, 0)).

Here, we relate the ELM theory to the context of AOS-ELM concept drift scenarios (see Table 1) as follows.

**Scenario 1 (virtual drift (VD)).**

Huang et al. [6] explained interpolation theory from ELM point of view as stated by the following description.

**Theorem  2.** *Given any small positive value ϵ* > 0*, any activation function which is infinitely differentiable in any interval, and N arbitrary distinct samples *(**x**_*i*_, **t**_*i*_) ∈ **R** ^*d*^ × *ℝ* ^*m*^ *, there exists L* < *N such that, for any input weight and bias pair *{**a**_*i*_, **b**_*i*_}_*i*=1_^*L*^ *randomly generated from any interval of* **R** ^*d*^ × *ℝ, according to any continuous probability distribution, with probability one, *‖**H** **β** − **T**‖ < *ϵ. Furthermore, if L* = *N, then with probability one, *‖**H** **β** − **T**‖ = 0.

According to Theorem  2 and Learning Principle I of ELM Theory [5], the input weight and bias as hidden nodes **H** parameters are independent of training samples and their learning environment through randomization. Their independence is not only in initial training but also in any sequential training stages. Thus, we can adjust the input weight and bias pair {**a**_*i*_, **b**_*i*_}_*i*=1_^*L*^ on any sequential stages and still make sure with probability one that ‖**H** **β** − **T**‖ < *ϵ*.

**Scenario 2 (real drift (RD)).**

Huang et al. [6] explained universal approximation capability of ELM as described by the following theorem.

**Theorem  3.** *Given any nonconstant piecewise continuous function g* : *ℝ*_*d*_ → *ℝ, if span *{*g*(**a**, **b**, **x**) : (**a**, **b**)*ℝ*_*d*_ × *ℝ*}* is dense in L2, for any continuous target function f and any function sequence *{*g*(**a**_*i*_, **b**_*i*_, **x**)}_*i*=1_^*L*^ *randomly generated according to any continuous sampling distribution, *lim_*L*→*∞*_‖*f* − *f*_*L*_‖ = 0* holds with probability one if the output weights* **β**_*i*_ *are determined by ordinary least square to minimize *‖*f*(**x**) − Σ_*L* *i*=1_ **β**_*i*_ *g*(**a**_*i*_, **b**_*i*_, **x**)‖.

Based on Theorem  3 and inspired by the related works [9, 14], we devised the AOS-ELM real drift capability by modifying the output matrix with zero block matrix concatenation to change the size dimension of the matrix without changing the value. Zero block matrix has meant the previous **β**_(*k*−1)_ has no knowledge about the new concept. ELM can approximate any complex decision boundary, as long as the output weights **β**_*i*_ are determined by ordinary least square to keep the minimum.

### 3.2. AOS-ELM Algorithms

In this section, we presented the AOS-ELM pseudocodes (the Matlab source code, data set, and demo file implementation are available at https://github.com/abudiman250172/adaptive-OS-ELM) in the *k*th sequential with **X**_(*k*)_ training input and **T**_(*k*)_ target to update *Model*_(*k*)_.

Basically, we have three pseudocodes, namely, OSELMSeq (Algorithm 1) as OS-ELM and CEOS-ELM pseudocodes; AOSELMVDSeq (Algorithm 2) as AOS-ELM pseudocodes tackling virtual drift; and AOSELMRDSeq (Algorithm 3) as AOS-ELM pseudocodes for addressing real drift. We can combine the pseudocodes together to form a hybrid drift Algorithm 4. We can increase the hidden nodes using CEOS-ELM in Algorithm 1 after AOSELMVDSeq or AOSELMRDSeq. For initialization, basically we can use any ordinary ELM initialization in offline learning mode.

**Figure 3:**
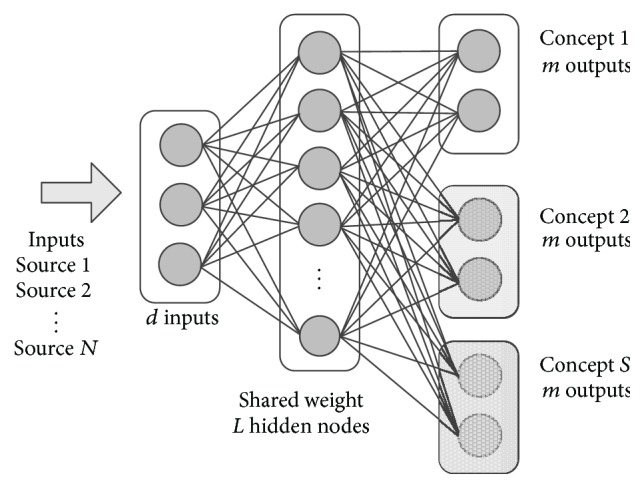
Output marginalization in AOS-ELM. The new block of output nodes assembled when the new concept *S* presented. Each concept has the same *m* output nodes quantity. Total output nodes become *S* × *m* output nodes.

For sudden drift scenario, we proposed output marginalization method by adding the new output nodes when the new concept presented (see Figure 3) and marginalized the output result by defining that **Y**_*s*_ class of concept *S* is =arg max_*y*_*s*__⁡**T**(*y*_*s*_). We scoped that the new concept has the same output nodes quantity with the previous concept. Output marginalization is by shifting the ELM output to the output nodes belonging to the new concept and ignoring the previous concept output nodes. This strategy is similar with classifier pruning in ELM ensemble. However, in output marginalization, we can reactivate the previous concepts by shifting back to the previous output nodes. If we want to forget the last concept totally, we can quickly delete the previous output nodes without impacting the generalization performance, or we can increase the hidden nodes at the same time with the drift event.

In regression, because we have only one output node, then we can employ sudden drift scenario by amplifying the related output node of the concept with a constant value that makes the maximum output **Y**_*s*_ approximated to 1.

The systematic rules make AOS-ELM more flexibe to handle complex consecutive drifts scenario. The AOS-ELM only stored the previous output weight **β**_*L*×*m*_ and autocorrelation **K**_*L*×*L*_. The autocorrelation **K** did not keep the training data. This makes AOS-ELM scalable for big streaming data without impacting the computation performance.

To improve the accuracy, we define the target values ∈{0,1}, so that **Y** class is =arg max_*y*_⁡**T**(*y*). According to [29], the target values ∈{0,1} are equivalent with ∈{−1,1}.

## 4. Experiments

### 4.1. Experiments Design in Classification

To verify our method, we designed some experiments with the following purposes:

- To investigate the effectiveness of AOS-ELM on tackling three concept drift scenarios (VD, RD, and HD) in two sequential patterns (sudden changes and recurring context). We used various data set starting with synthetic data set (SEA, STAGGER) and then with real data set in handwritten recognition (MNIST, USPS). Each data set has different drift characteristics. This experiment is presented in Sections 4.2 and 4.4. We also demonstrated the AOS-ELM capability as drift detection role in Section 4.3 using SEA data set.
- To investigate the effectiveness of AOS-ELM on transfer learning to combine different data set sources. This experiment is presented in Section 4.4 using two data set sources (MNIST and USPS) in handwritten recognition problem.
- To investigate the effect of hidden nodes increase in the drift events and how it impacts performance. This experiment is presented in Section 4.5.

We used Matlab*™* running on Microsoft Windows*™* Computer with 4-core 2.5 GHz processor and 8 GB memory.

**Figure 4:**
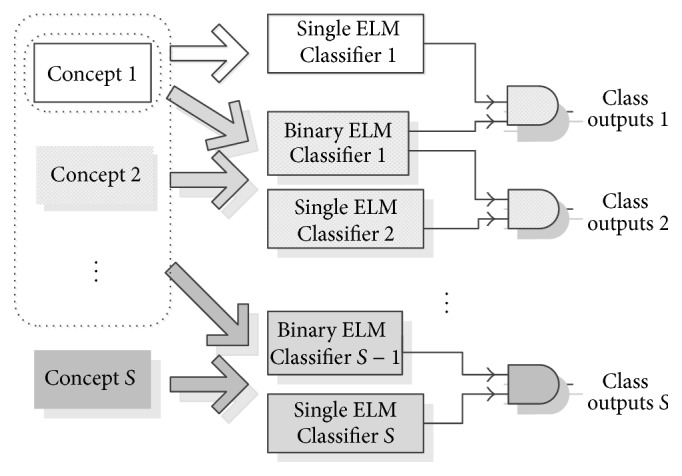
Hierarchical ELM ensemble for MNIST + USPS experiment. The gray shadow showed the new classifiers assembled when the new concept presented.

Our experiments are organized as follows:

(1) Simulation benchmark tests on the data sets commonly used in concept drift handling of stream data, for example, SEA [19] and STAGGER [20] (see Table 2(a)). Both data sets are binary classification problem. SEA has 3 inputs with random integer values from 0 to 9. STAGGER has three inputs with multiple category values from 1 to 3 (total inputs are 9). SEA and STAGGER are the examples of concept drift caused by discriminant function changes while the number of attributes and classes from all concepts is still the same. The change type is sudden drift. The expected result is that the classifier has good performance for the newest concept [22].
(2) We tested our algorithm with real-world public data sets from MNIST numeric (0 to 9) [17] and the USPS alphanumeric (A to Z, 0 to 9) handwritten data set [18]. We used original grey-level image attributes [*X*_grey_] of MNIST data set and the combination of [*X*_grey_] with additional attributes from the 9 × 9 bins histogram of orientated gradients (*X*_HOG_) of grey-level image features [30]. For USPS, we added more data with Gaussian random and salt-pepper noises. Refer to Table 2(a) for detailed data set information.
(3) We designed the initial input weights and bias based on robust OS-ELM with regularization scalar *c* (ROS) [31] and then based on initial random from the normal distribution (NORM). The activation function is sigmoid. The pseudoinverse function is the orthogonal projection using ridge regularization.
(4) Let us define the following concept as
  (i) **C**_1_ is MNIST[*X*_grey_] class (1–6),
  (ii) **C**_2_ is MNIST[*X*_grey_] class (7–10),
  (iii) **C**_3_ is MNIST[*X*_grey_ *X*_HOG_] class (1–6),
  (iv) **C**_4_ is MNIST[*X*_grey_ *X*_HOG_] class (7–10),
  (v) **C**_5_ is MNIST[*X*_grey_ *X*_HOG_] class (1–10),
  (vi) **C**_6_ is USPS[*X*_grey_ *X*_HOG_] class (1–10, A–Z).
  We followed the simulated concept drift methods in Dries and Rückert [32]. We simulated sudden drift by splitting the composition into two groups, for example, **C**_1_ and **C**_2_, and recurring context by shuffling the composition of **C**_1_ and **C**_2_. We set the sequential training flow to be the following drift equation:
  (a) Experiment 1: virtual drift: $MNIST[X_{\text{grey}}]\;\begin{array}{c} ⋙\\ \text{VD} \end{array}MNIST[X_{\text{grey}}X_{\text{HOG}}]$,
  (b) Experiment 2: real drift: for recurring context, $C_{1}\begin{array}{c} ⋙\\ \text{RD} \end{array}\text{shuffled}(C_{1},C_{2})$; for sudden drift: $C_{1}\begin{array}{c} ⋙\\ \text{RD} \end{array}C_{2}$,
  (c) Experiment 3: hybrid drift: $C_{1}\begin{array}{c} ⋙\\ \text{HD} \end{array}\text{shuffled}(C_{3},C_{4})$,
  (d) Experiment 4: MNIST + USPS transfer learning: $C_{5}\begin{array}{c} ⋙\\ \text{RD} \end{array}C_{6}$.
(5) We measured the performance based on Table 2(c). The testing accuracy and Cohen's Kappa are to show the quantitative measurement. The predictive accuracy is to demonstrate the trend in a line chart. The sudden drift performance is based on the forgetting capability that compared the testing accuracy of the latest concept against all the previous concepts.
(6) We compared the AOS-ELM performance with nonadaptive online sequential and offline version of ELM classifier. The performance expectation of sequential version classifier is to approximate the offline version of the classifier (desiderata for online classifiers [22]). We also compared with adaptive ELM ensemble method (see Figure 4). We designed the hierarchical ensemble using two models of ELM classifier with different roles (see Figure 4). The first role is a binary classifier that acts as a director based on one against all (OAA) classification. The binary classifier needs all sequential training data to be recalled (full memory). Another role is the data classifier. This ensemble requires total 2*S* − 1 classifiers for *S* concepts, thus, not effective for consecutive concept drift case, for example, SEA concepts. The ensemble also applied outdated classifier pruning when the ensemble detects that the previous attributes need to be replaced.

### 4.2. SEA and STAGGER Concepts Result

We addressed the question whether nonadaptive OS-ELM and CEOS-ELM with *δL* increase could handle the concept drift situation. We compared between AOS-ELM with no *δL* increase (AOS-ELM1) and with *δL* increase (AOS-ELM2). We used 5-fold cross-validation and compared between NORM and ROS parameter. For SEA, parameters *L*_0_ = 3000 and *δL* = 500 increase per drift. For STAGGER, parameters *L*_0_ = 9 and *δL* = 5 hidden nodes increase per drift.

**Figure 5:**
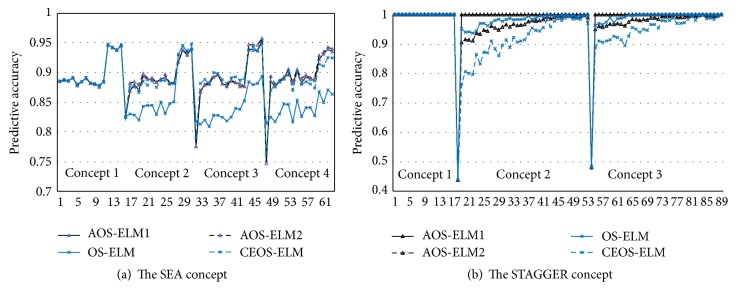
Predictive accuracy of AOS-ELM1 (black line △) and AOS-ELM2 (black dashes △) compared with OS-ELM (blue line ×) and CEOS-ELM (blue dashes  ×).

The AOS-ELM has better accuracy with better recovery time (see Tables 3(a) and 3(b)) than CEOS-ELM, whereas nonadaptive OS-ELM fails (see Figure 5). The AOS-ELM2 improved the forgetting capability better than AOS-ELM1. In comparison with Kolter and Maloof result using dynamically weighted majority (DWM) of naive Bayes (DWM-NB) for SEA, AOS-ELM result is near to the DWM result. Comparison with inducing decision trees (DWM-ITI) for STAGGER [20], AOS-ELM outperformed DWM (see Tables 3(a) and 3(b)).

### 4.3. Concept Drift Detection

**Figure 6:**
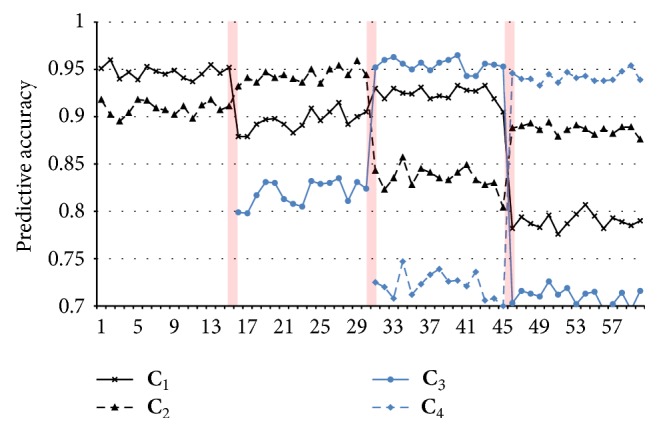
Predictive accuracy of AOS-ELM in SEA for each concept with *m* output (see Figure 3). We can consider the intersection point between accuracy decrease of previous concept and accuracy increase of current concept as change point (displayed as thin vertical shadow line).

The drift detection works based on loss estimation (see Figure 2) that compared current prediction accuracy with the previous feedback. Using similar method on [33, 34], we can evaluate the intersection point between accuracy decrease and increase in Figure 6. If the consecutive loss performance exceeded a certain threshold, then drift warning status is triggered. We measured the output performance from the new concept output and compared with the previous output. If it met certain criteria, then the new AOS-ELM is committed. Otherwise, the previous AOS-ELM is rolled back.

### 4.4. MNIST and MNIST + USPS Result

We measured the testing accuracy based on holdout test data by 10x experiment trials. The results are as follows.

**Experiment 1 (virtual drift).**

The AOS-ELM of [*X*_grey_ *X*_HOG_] has Cohen's kappa of testing accuracy 95.72 (0.21)% approximated to its nonadaptive ELM and offline ELM of [*X*_grey_ *X*_HOG_] version with the same hidden nodes number *L* = 2000. It has better accuracy than single attribute [*X*_grey_] or [*X*_HOG_] only (see Table 4(b)). It proves our explanation in the theoretical background on Section 3.1.

*Note*. We set *L*_0_ = 200 for [*X*_HOG_] ELM based on the same ratio between number of input nodes with hidden nodes of [*X*_grey_] ELM.

**Experiment 2 (real drift).**

The final result is shown in Table 5(b): the AOS-ELM has better Cohen's kappa performance for all concepts than ELM ensemble and little exceeds its nonadaptive and offline ELM (Table 5(b)).

As in the split composition, the AOS-ELM with *δL* increase has better performance in forgetting capability than the AOS-ELM with no *δL* increase (see Table 8(b)).

**Experiment 3 (hybrid drift).**

The final result is shown in Table 5(c): the AOS-ELM has better Cohen's kappa performance for HD than ELM ensemble and approximates to its nonadaptive and offline ELM.

**Experiment 4 (MNIST + USPS transfer learning).**

**Figure 7:**
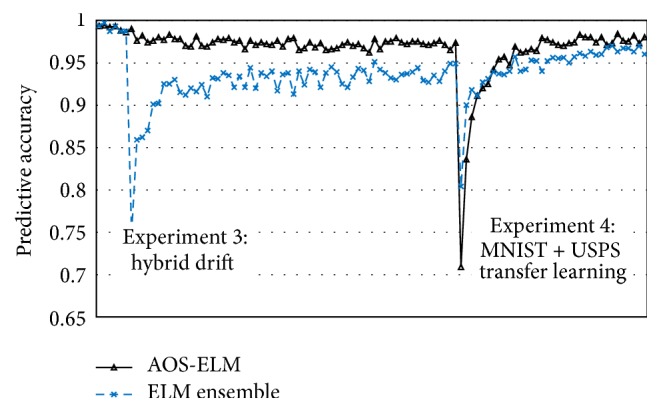
Predictive accuracy of AOS-ELM (black line) over sequential data for MNIST + USPS compared with ELM ensemble (blue dash line).

The AOS-ELM has better Cohen's kappa performance for both numeric and alphabet concepts than ELM ensemble (see Table 5(d)) and approximates to its nonadaptive and offline ELM. The AOS-ELM shows better recovery time than ELM ensemble in Figure 7.

### 4.5. The Effect of Hidden Nodes Increase

The initial size of hidden nodes *L*_0_ selection is important to have good generalization performance. Researches [3, 6] suggested for the hidden nodes size to be at minimum equal to the rank value of training data. However, in a data stream, it is hard to determine a fixed number of hidden nodes following that suggestion. The larger *L*_0_ requires more computation resources and processing time, probably not giving a significant result at the end. Thus, we have a requirement to increase *δL* in sequential stage [9].

The experiment result in Table 6 shows that the performance improved when certain hidden nodes size increases. We used different initial hidden nodes size (*L*_0_) condition: 2000, 666 (the rank value of initial training data), and 713 (the rank value of total training data). We used also different conditions of hidden nodes increase (*δL*) by using ROS parameters on the drift event: 0 (no increase), 500, 1000, and 2000. However, the larger *L*_0_ has better influence than *δL* increase.

We studied the effect of hidden nodes increased in the sequential phase as follows.

*(1) “Underfitting” Condition*. “Underfitting” is the condition when the model does not fit the data well enough which makes nonconvergence. Based on an empirical experiment with *δL* increase in the sequential phase on Table 7, we investigated particular condition when the AOS-ELM classifier has a bad result. We realized that the ELM performance is dependent upon finding general matrix inverse of **H**. Based on orthogonal projection method in CEOS-ELM, we can employ the rank value of $\hat{P}$ as evaluation parameter to detect “underfitting”.

The $\hat{P}$ is approximation to matrix (**H** ^T^ **H**)^−1^. The full rank of **P**_*L*×*L*_ is ideally equal to *L*. However, certain condition in the sequential training, for example, poor training data or poor learning parameter selection, may cause the diagonal squared matrix **P** to be less diagonalizable [35], thus not full rank anymore.

In the sequential learning, we can compare $\text{Rank}(\hat{P})$ before and after hidden nodes increase. The expected result is positive increment. If the rank value becomes lower after hidden nodes increase, then it has a higher probability for “underfitting” condition to occur. $\text{Rank}(\hat{P})$ is determined by the block size of training data, the number of hidden nodes increment, the *c* scalar selection in ROS parameter, the activation function, input weight, and bias random assignment method. In this experiment, we focused on the block size, the number of hidden nodes, and *c* scalar selection. Using $\text{Rank}(\hat{P})$ as evaluation parameter is more efficient because we do not need to compute **β**.

*(2) Sudden Drift*. In Tables 8(a) and 8(b), the hidden nodes increase can improve the forgetting capability on sudden drift (it reduced the accuracy of the outdated concept).

In CEOS-ELM, when *δL* increases in the same time with drift, it makes $\left[\begin{array}{cc} H_{1} & 0\\ H_{2} & \Delta H_{2} \end{array}\right]$ and the new concept target $\left[\begin{array}{c} 0\\ t_{2} \end{array}\right]\in T_{2}$ in split composition, while previous concept $\left[\begin{array}{c} t_{1}\\ 0 \end{array}\right]\in T_{1}$. Thus, in the process of finding $\hat{\beta }$ it becomes simplified because [**H**_2_Δ**H**_2_] is partially trained by **t**_2_ only and not by **t**_1_. Thus, it reduced the generalization capability of [**H**_2_Δ**H**_2_] to recognize **T**_1_ problem.

## 5. AOS-ELM in Regression

We can use the similar real drift scenario with output marginalization and output amplification to solve concept drift problem in regression. In this experiment, we used AOS-ELM with single input node and single output node per concept. We defined the following concept as

(i) **C**_1_ is* sinc* function with 50000 training/5000 testing,
(ii) **C**_2_ is* sinus* function with 50000 training/5000 testing,
(iii) **C**_3_ is* gaussian* function with 50000 training/5000 testing.

The sequential experiments are following drift equations:

(1) For Experiment 1, $C_{1}\begin{array}{c} ⋙\\ \text{RD} \end{array}C_{2}$,
(2) For Experiment 2, $C_{1}\;\begin{array}{c} ⋙\\ \text{RD} \end{array}\;C_{2}\;\begin{array}{c} ⋙\\ \text{RD} \end{array}\;C_{3}$.

**Figure 8:**
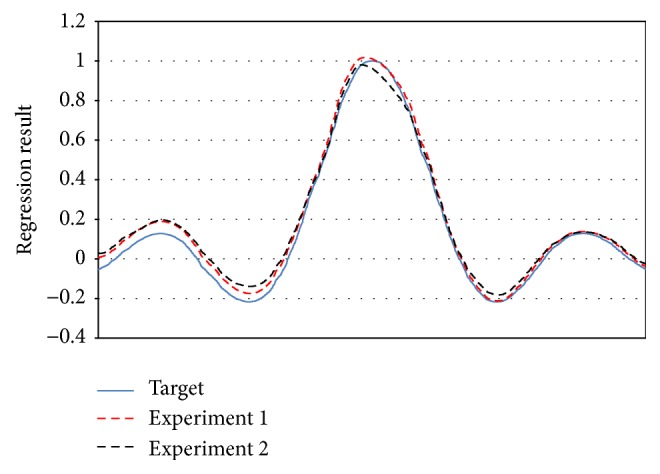
Regression result of **C**_1_ for Experiment 1 (red dash line) using constant value 4 and Experiment 2 (black dash line) using constant value 8.25.

**Figure 9 fig9:**
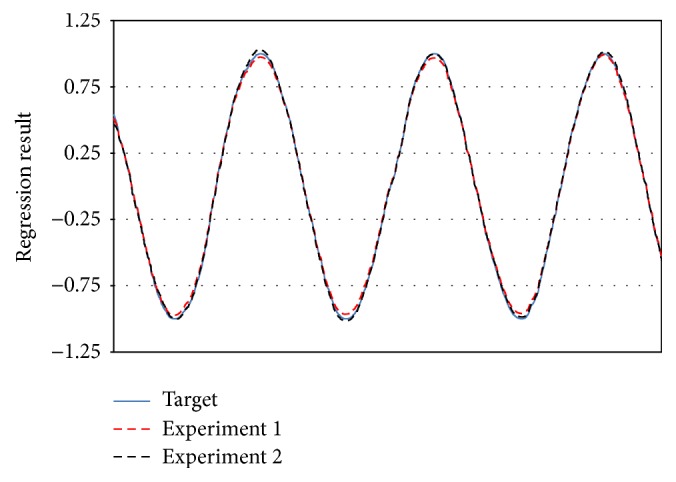
Regression result of **C**_2_ for Experiment 1 (red dash line) using constant value 1.3 and Experiment 2 (black dash line) using constant value 3.

**Figure 10:**
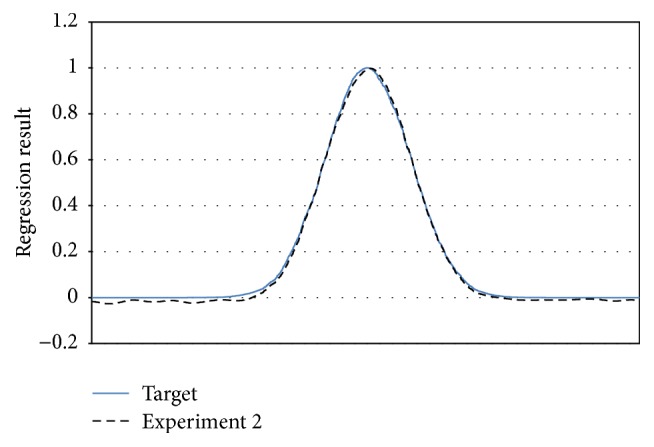
Regression result of **C**_3_ for Experiment 2 (black dash line) using constant value 1.85.

We presented the result on Figures 8 [Fig fig9]–10 to compare the performance of each concept at the end of each training experiment. Our objective is to show the AOS-ELM regression capability to keep the previous regression concept knowledge. We select the constant value giving the best regression result of each concept. The AOS-ELM has *L*_0_ = 100, *δL* = 0, and *sigmoid* function. More drifts occurring will weaken the older concepts. Thus, the AOS-ELM needs larger amplifier constant value.

## 6. Simulation in Big Data Stream: Intrusion Detection System (IDS) KDD Cup 1999

IDS is a network security technology that scans any network packet traffic to detect any potential exploits, then sending the alarm or taking some active action to Intrusion Prevention System. Some machine learning methods have been applied with the hope of improving detection rates and adaptive capability [36].

**Figure 11:**
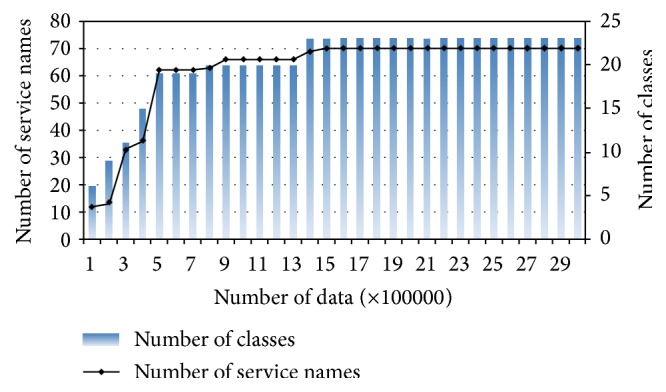
The changes on service names and number of classes following the streaming data.

In this experiment, we used KDD Cup 1999 Competition data set. The full data set had 4898431 network packets grouped to be 23 classes (One normal class and 22 attack names based on a signature-based detection) [37]. The data set has a control information (CI) header for delivering the data in numerical and multicategorical values as features. We focused on service names (IP ports) attributes because they are specific differentiators for applications. The CI and the number of attack classes are not stationary. We analyzed the data set for the growing of service names and the number of class attack in the whole data set on Figure 11. The challenge in IDS data set is imbalanced data between the classes. The highest number of data is for “normal” class, and the lowest number is for “spy” class (only 2 packets). To simplify the experiment, we use oversampling by adding more data based on the random normal distribution of packet signatures and under sampling approaches by dropping some samples randomly.

Based on the growing of service names and the number of classes analysis (see Figure 11), we designed one drift scenario based on two concepts (Table 9(b)). **C**_1_ has ten classes and 37 service names and **C**_2_ has 23 classes and 70 service names. Total training data for each concept is 920000 packets. There is no data repetition from the previous event, except at the end of **C**_2_ sequential training. The composition between **C**_1_/**C**_2_ on HD event is 230000/690000. The validation data set of **C**_2_ is selected from all packets from minority classes and randomly selected original majority classes (10422 packets). We used holdout method with 5x trials. We used AOS-ELM1 for *δL* = 0 and AOS-ELM2 for *δL* = 500 (other ELM parameters are same: *L*_0_ = 1000, NORM,* sig*). The AOS-ELM result in this experiment can approximate the nonadaptive OS-ELM on **C**_2_ (see Table 9(b)).

## 7. Challenges and Future Research

Based on AOS-ELM experiments, we face some challenges, which are as follows:

- We need to investigate the optimum transition space that minimizes the gap to the new concept learning model. In certain case, the AOS-ELM may have the “underfitting” condition and require larger training data to achieve the new convergence.
- We need to check the consistency of AOS-ELM for different pseudoinverse methods (e.g., Greville's method [23]).

We suggest some ideas for AOS-ELM future researches as follows:

- The need for transfer learning to solve big data problem when the distribution data changes.
- The AOS-ELM integration with other ELM methods, for example, Weighted OS-ELM for imbalanced learning [38], ELM Autoencoder (ELM-AE) [39], and Stacked ELM [40].
- A detailed systematic explanation based on rule extraction [41] for AOS-ELM in handling adaptive environment.

## 8. Conclusion

The proposed method gives better adaptive capability than nonadaptive OS-ELM and CEOS-ELM in terms of retaining the recognition performance when handling concept drifts. It uses a simple line of code easy to deploy especially for consecutive drifts, compared with adaptive ensemble methods. While most adaptive classifiers work differently for each of virtual, real drift, and hybrid drift scenarios, the AOS-ELM tackles those drifts through simple block matrix reconstruction and rank evaluation.

AOS-ELM satisfied the requirement criteria in terms of accuracy, simplicity, speed, and flexibility. However, in certain VD and HD cases, the AOS-ELM accuracy may not exceed the nonadaptive sequential ELM, which include the future training data. In RD cases, the AOS-ELM has better accuracy. In a real data implementation, the nonadaptive ELM is better and preferred when we know exactly the future behavior of data. However, we can not predict it precisely. We believe using larger training data, the AOS-ELM performance will approximate the expected value of nonadaptive sequential ELM or offline ELM, which use the future training data. The AOS-ELM can also add learning adaptation function to the previous offline learning model. It makes AOS-ELM an excellent choice for the unpredictable situation.

The AOS-ELM tackles sudden drift change type as well as recurrent context change type. The output marginalization strategy is implemented by simply shifting the output nodes belonging to the latest concept. The AOS-ELM does need to increase the hidden nodes to improve the forgetting capability for sudden drift change type. To make sure of the convergence to the expected learning model, we proposed the rank value of the pseudoinverse autocorrelation hidden nodes matrix as evaluation parameter to prevent “underfitting” condition that makes the accuracy performance dropped.

We can consider the AOS-ELM as another type of ELM ensemble formation using shared and interconnected hidden nodes between ensemble members. We can implement the AOS-ELM in similar fashion compared to the ELM ensemble for adaptive learning scheme, but with better performance, simplicity, and more resource efficiency. However, the AOS-ELM does have some drawbacks. Any hidden node changes could impact all notions.

## Figures and Tables

**Algorithm 1:**
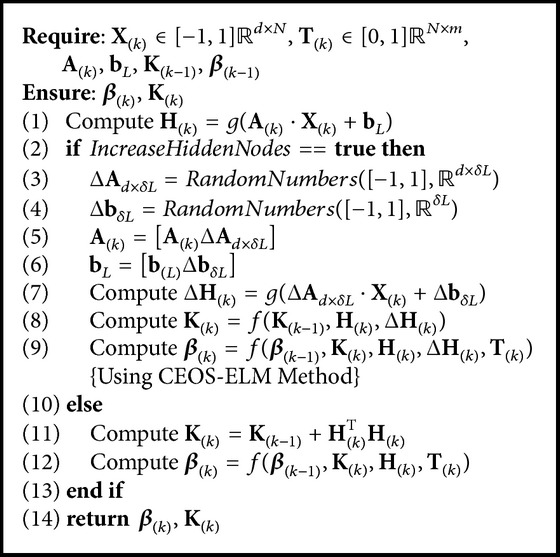
Algorithm OSELMSeq {OS-ELM sequential}.

**Algorithm 2:**
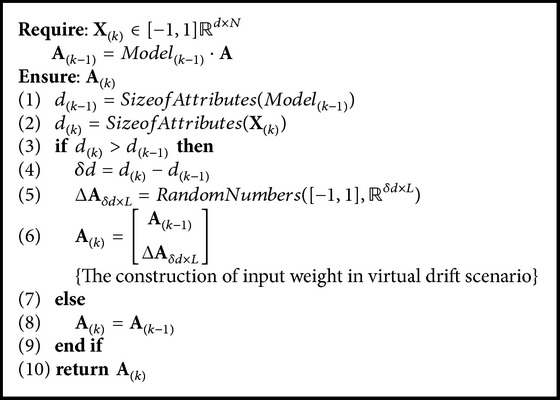
Algorithm AOSELMVDSeq {AOS-ELM sequential-virtual drift}.

**Algorithm 3:**
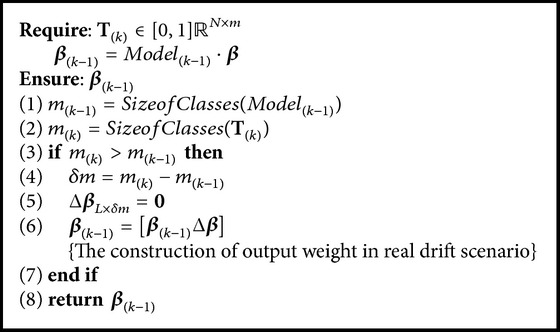
Algorithm AOSELMRDSeq {AOS-ELM sequential-real drift}.

**Algorithm 4:**
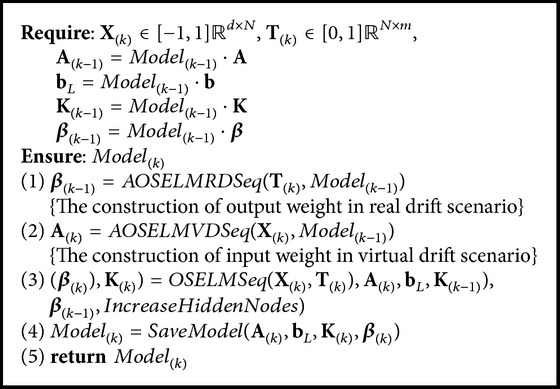
Algorithm AOS-ELM sequential-hybrid drift.

**Table:** (a) The experiment design scenarios

Data set	Virtual drift	Real drift	Hybrid drift	Compared methods
SEA	—	✓	—	OS-ELM, CEOS-ELM, Kolter [20]
STAGGER	—	✓	—	OS-ELM, CEOS-ELM, Kolter [20]
MNIST	✓	✓	✓	OS-ELM, Offline ELM, ELM ensemble
MNIST + USPS	—	✓	✓	OS-ELM, offline ELM, ELM ensemble

**Table:** (b) Concept drift sequential patterns

Data set	Sequential patterns scenarios	Cause of shift
SEA	Sudden change	Linear discriminant function
STAGGER	Sudden change	Logical discriminant rule
MNIST	Sudden change and recurring context	Additional attributes or classes
USPS	Recurring context	Additional attributes or classes

**Table:** (a) Data set dimension and quantity

Data set	Concepts	Inputs	Outputs	Quantity (×concepts)
SEA	4	3	2	20000 (×4)
STAGGER	3	9	2	4400 (×3)
MNIST	2	784, 865	10	70000 (×2)
USPS	1	865	36	48908 (×1)

**Table:** (b) Evaluation method

Data set	Evaluation method	Training	Testing
SEA	5-fold cross-validation	16000 (×4)	4000 (×4)
STAGGER	5-fold cross-validation	3520 (×3)	880 (×3)
MNIST	Holdout (10x trials)	60000 (×2)	10000 (×2)
USPS	Holdout (10x trials)	35050	13858

**Table:** (c) Performance measurements

Measure	Specification
Accuracy	The accuracy of classification in % from # correctly classified/# total instances
Predictive accuracy	The accuracy measurement of the future sequential training data [20]
Testing accuracy	The accuracy measurement of the testing data set excluded from the training
Forgetting capability	The testing accuracy differences between the current concept with the previous concepts
Cohen's kappa and kappa error	The statistic measurement of interrater agreement for categorical items

**Table:** (a) Testing accuracy in % for SEA with **C**_1__RD_^⋙^ **C**_2__RD_^⋙^ **C**_3__RD_^⋙^ **C**_4_

Method	Parameter	**C**_1_	**C**_2_	**C**_3_	**C**_4_
OS-ELM	NORM	89.48 ± 0.33	86.47 ± 0.37	83.41 ± 0.93	85.32 ± 0.45
	ROS	89.51 ± 0.37	86.43 ± 0.38	83.49 ± 0.95	85.31 ± 0.49

CEOS-ELM	NORM	82.40 ± 0.27	89.33 ± 0.43	75.80 ± 0.87	90.30 ± 0.35
	ROS	84.54 ± 0.59	89.96 ± 0.59	77.97 ± 1.05	90.26 ± 0.69

*AOS-ELM1*	NORM	89.84 ± 0.28	90.03 ± 0.25	89.67 ± 0.61	**90.33** ± 0.39
	ROS	89.76 ± 0.34	90.02 ± 0.25	89.76 ± 0.55	**90.34** ± 0.38

*AOS-ELM2*	NORM	50.58 ± 1.18	50.71 ± 1.19	48.50 ± 10.10	**90.34** ± 0.30
	ROS	65.78 ± 1.21	65.67 ± 1.19	64.09 ± 1.89	**90.14** ± 1.34

**Table:** (b) Testing accuracy in % for STAGGER with **C**_1__RD_^⋙^ **C**_2__RD_^⋙^ **C**_3_

Method	Parameter	**C**_1_	**C**_2_	**C**_3_
OS-ELM	NORM	51.89 ± 3.48	81.61 ± 4.74	67.18 ± 5.80
	ROS	49.77 ± 1.96	84.16 ± 1.61	66.93 ± 2.00

CEOS-ELM	NORM	21.98 ± 1.57	53.66 ± 4.34	97.84 ± 4.32
	ROS	23.23 ± 1.93	52.11 ± 2.35	99.27 ± 1.45

*AOS-ELM1*	NORM	97.64 ± 1.95	**100.00** ± 0.00	**100.00** ± 0.00
	ROS	**100.00** ± 0.00	**100.00** ± 0.00	**100.00** ± 0.00

*AOS-ELM2*	NORM	59.66 ± 5.65	70.91 ± 10.93	**100.00** ± 0.00
	ROS	56.20 ± 9.56	69.41 ± 14.05	**100.00** ± 0.00

**Table:** (a) Benchmark result, nonadaptive OS-ELM and offline ELM

Performance	ELM method	[*X*_grey_] (*L* = 2000)	[*X*_HOG_] (*L* = 200)	[*X*_grey_ *X*_HOG_] (*L* = 2000)
Testing accuracy	OS-ELM	95.32 ± 0.12	94.64 ± 0.15	**96.86** ± 0.13
	Offline ELM	95.33 ± 0.13	94.66 ± 0.15	**96.85** ± 0.06

Cohen's kappa	OS-ELM	94.80 (0.24)	94.04 (0.25)	**96.51 (0.19)**
	Offline ELM	94.81 (0.23)	94.06 (0.25)	**96.50 (0.19)**

**Table:** (b) VD experiment, AOS-ELM (*L* = 2000)

Drift	Testing accuracy	Cohen's kappa
MNIST [*X*_grey_] $\begin{array}{c} ⋙\\ VD \end{array}$ MNIST [*X*_grey_ *X*_HOG_]	**96.15** ± 0.08	**95.72 (0.21)**

**Table:** (a) Benchmark result, nonadaptive OS-ELM and offline ELM

Source	Class	Testing accuracy		Cohen's kappa	
		OS-ELM	Offline ELM	OS-ELM	Offline ELM
MNIST [*X*_grey_]	(1–6)	95.99 ± 0.15	96.00 ± 0.14	95.21 (0.30)	95.22 (0.30)
	(7–10)	94.30 ± 0.22	94.32 ± 0.19	92.50 (0.48)	92.53 (0.48)

MNIST [*X*_grey_ *X*_HOG_]	(1–6)	97.59 ± 0.11	97.49 ± 0.09	97.10 (0.23)	97.00 (0.24)
	(7–10)	95.76 ± 0.26	95.87 ± 0.12	94.40 (0.42)	94.55 (0.42)

MNIST + USPS [*X*_grey_ *X*_HOG_]	(1–10)	96.01 ± 0.10	96.08 ± 0.08	95.56 (0.02)	95.65 (0.02)
	(A–Z)	99.94 ± 0.02	99.94 ± 0.02	99.94 (0.02)	99.93 (0.02)

**Table:** (b) RD experiment, ELM ensemble (3 classifiers, full memory) versus AOS-ELM

Source	Concept	Testing accuracy		Cohen's kappa	
		ELM ensemble	AOS-ELM	ELM ensemble	AOS-ELM
MNIST [*X*_grey_]	**C**_1_ (1–6)	94.58 ± 0.17	**96.09** ± 0.12	93.54 (0.35)	**95.10 (0.31)**
	**C**_2_ (7–10)	91.60 ± 0.29	**94.34** ± 0.16	89.04 (0.57)	**92.56 (0.48)**

**Table:** (c) HD experiment, ELM ensemble (3 classifiers, full memory, outdated classifier pruning) versus AOS-ELM

Source	Concept	Testing accuracy		Cohen's kappa	
		ELM ensemble	AOS-ELM	ELM ensemble	AOS-ELM
MNIST [*X*_grey_]	**C**_3_ (1–6)	94.48 ± 0.33	**97.01** ± 0.18	93.42 (0.35)	**96.42 (0.26)**
MNIST [*X*_grey_ *X*_HOG_]	**C**_4_ (7–10)	92.29 ± 0.36	**96.05** ± 0.19	89.95 (0.55)	**94.78 (0.40)**

**Table:** (d) MNIST + USPS experiment, ELM ensemble (5 classifiers, full memory, outdated classifier pruning) versus AOS-ELM

Source	Concept	Testing accuracy		Cohen's kappa	
		ELM ensemble	AOS-ELM	ELM ensemble	AOS-ELM
MNIST [*X*_grey_ *X*_HOG_]	**C**_5_ (1–10)	88.17 ± 11.06	**95.91** ± 0.12	86.94 (0.33)	**95.46 (0.22)**
USPS [*X*_grey_ *X*_HOG_]	**C**_6_ (A–Z)	99.80 ± 0.05	**99.95** ± 0.03	99.79 (0.40)	**99.95 (0.02)**

**Table 6:** Testing accuracy in Cohen's kappa (kappa error) in % AOS-ELM MNIST for different *L*_0_ and *δL*.

Scenario	*L*_0_	*δL* = 0	*δL* = 500	*δL* = 1000	*δL* = 2000
VD	2000	95.92 (0.21)	96.37 (0.20)	96.83 (0.18)	**96.89 (0.18)**
	666	93.10 (0.27)	95.18 (0.23)	**96.18 (0.20)**	95.60 (0.22)
	713	93.30 (0.26)	95.31 (0.22)	**96.28 (0.20)**	96.09 (0.20)

RD	2000	94.71 (0.24)	94.93 (0.23)	95.39 (0.22)	**95.42 (0.22)**
	630	91.3 (0.30)	91.67 (0.29)	93.61 (0.26)	**94.04 (0.25)**
	713	91.71 (0.29)	92.70 (0.28)	93.82 (0.25)	**94.23 (0.25)**

**Table 7:** Predictive accuracy performance in AOS-ELM for MNIST using different parameters and $Rank(\hat{P})$ before and after *δL* increase. Each experiment is repeated 100x trials to get the probability of predictive accuracy ≤ 50%.

Scenario	*L*_0_	*δL*	Batch size	*c*	Before	After	Pred. acc. ≤ 50%
RD	630	500	1000	10	630	1130	0%
	630	500	500	10	1130	1122	**7**%
	630	500	100	10	1130	614	**5**%
	630	500	10	10	640	640	0%
	630	100	1000	10	630	730	0%
	630	100	500	10	730	730	0%
	630	100	100	10	730	730	0%
	630	100	10	10	640	640	0%
	2000	1000	100	5	2000	1868	**3**%
	2000	1000	100	1	2000	1947	**16**%
	2000	1000	100	0.5	2000	1946	**17**%
	2000	1000	100	0.05	2000	2100	0%

VD	666	500	500	5	666	1166	0%
	666	500	100	5	666	1166	0%
	666	100	500	5	666	766	0%
	666	100	100	5	666	766	0%
	666	50	500	5	666	716	0%
	666	50	100	5	666	716	0%

**Table:** (a) Sudden drift effect caused by split training composition with hidden nodes *δL* increase

Data	AOS-ELM	*δL*	Concept	Testing accuracy
SEA	*L*_0_ = 3000	0	**C**_2_	**90.00** ± 0.59
			**C**_4_	**90.24** ± 0.61
		500	**C**_2_	66.29 ± 1.12
			**C**_4_	**90.12** ± 0.52

MNIST	*L*_0_ = 2000	0	**C**_1_	**96.42** ± 0.21
			**C**_2_	**93.68** ± 0.23
		500	**C**_1_	17.59 ± 0.98
			**C**_2_	**97.08** ± 0.15

**Table:** (b) MNIST RD simulation: the effect of hidden nodes *δL* increase for split and shuffled training composition (*L*_0_ = 2000)

Data	*δL*	Composition	**C**_1_	**C**_2_
MNIST	0	Split	**96.42** ± 0.21	**93.68** ± 0.23
		Shuffled	**96.09** ± 0.12	**94.34** ± 0.16
	500	Split	17.59 ± 0.98	**97.08** ± 0.15
		Shuffled	**96.53** ± 0.12	**94.29** ± 0.25
	1000	Split	8.65 ± 1.13	**97.64** ± 0.18
		Shuffled	**96.74** ± 0.14	**94.78** ± 0.10

**Table:** (a) Benchmark result, nonadaptive OS-ELM

Concept	Parameters	Testing accuracy	Cohen's kappa
**C**_1_	OS-ELM	50.87 ± 0.01	45.81 (0.54)
**C**_2_	OS-ELM	94.58 ± 0.05	94.03 (0.24)

**Table:** (b) Performance result on the drift event, AOS-ELM

Drift	Parameters	Testing accuracy	Cohen's kappa
$C_{1}\begin{array}{c} ⋙\\ HD \end{array}C_{2}$	AOS-ELM1	92.18 ± 2.73	91.38 (0.29)
$C_{1}\begin{array}{c} ⋙\\ HD \end{array}C_{2}$	AOS-ELM2	94.64 ± 0.06	94.10 (0.24)
End of full **C**_2_	AOS-ELM1	93.45 ± 1.18	92.78 (0.27)
End of full **C**_2_	AOS-ELM2	94.57 ± 0.11	94.02 (0.25)
